# A Landsat-based vegetation trend product of the Tibetan Plateau for the time-period 1990–2018

**DOI:** 10.1038/s41597-019-0075-9

**Published:** 2019-05-31

**Authors:** Fabian Ewald Fassnacht, Christopher Schiller, Teja Kattenborn, Xinquan Zhao, Jiapeng Qu

**Affiliations:** 1 Institute of Geography and Geoecology, Karlsruhe Institute of Technology, Kaiserstraße 12, 76131 Karlsruhe, Germany; 2 Key laboratory of adaptation and evolution of plateau biota, Northwest Institute of Plateau Biology, Chinese Academy of Sciences, Qinghai, 810008 China; 3 Qinghai Provincial Key Laboratory of Restoration Ecology in Cold Region, Qinghai, 810008 China

**Keywords:** Grassland ecology, Restoration ecology

## Abstract

The Tibetan Plateau is a unique, biodiverse ecosystem with an important role in the climate and hydrological system of Asia. Its vegetation supports important functions including fodder provision, erosion prevention and water retention. Assessing vegetation trends of the Tibetan Plateau is crucial to understand effects of recent climate and land-use changes. Most existing vegetation trend products covering the entire Tibetan Plateau have a coarse spatial grain and cover short temporal ranges. This hampers their applicability in studies conducted at local scales where land-use decisions take place and at time scales where climate changes become apparent. Here, we present vegetation trend products for the entire Tibetan Plateau at a spatial resolution of 30 m for the time period 1990–2018. These products include results of a modified Mann-Kendall trend test applied to annual Landsat-based NDVI mosaics, composed from all satellite observations acquired during the vegetation periods as well as NDVI difference images. These data can be valuable to many researchers including for example wildlife ecologists, rangeland experts and climate change researchers.

## Abstract (summary)

**Table:** 

Design Type(s)	modeling and simulation objective • data transformation objective • time series design
Measurement Type(s)	vegetation layer
Technology Type(s)	computational modeling technique
Factor Type(s)	geographic location • temporal_interval
Sample Characteristic(s)	Tibetan Plateau • plateau

Machine-accessible metadata file describing the reported data (ISA-Tab format)

## Background & Summary

The Qinghai Tibetan Plateau (QTP) is the highest plateau ecosystem of the world. It harbours a range of ecosystems including forests, grasslands and shrublands^1^. The QTP provides crucial ecosystem services for humanity including the provision of water to about 40% of the Earth’s population^2^. Due to its high elevation, it serves as local heat source in the atmosphere with effects on the general circulation system and particularly on India’s Monsoon system^3^. The QTP is further known for its high level of biodiversity^4^ including high proportions of endemic species (e.g., Tibetan antelope, wild yak, snow leopard) which according to current knowledge co-existed for millennia with the traditional nomadic pastoral system and the Tibetan culture^5,6^.

A key parameter of the QTP system is its vegetation composition and cover. For example, vegetation cover drives the albedo and transpiration rates of the Plateau surface and hence influences energy-fluxes between surface and atmosphere^7^. Large-scale loss or gain in vegetation cover will change the amount of directly reflected electromagnetic radiation with potential effects on climate systems. A degraded vegetation-cover also leads to a reduced water retention capability^8^ and may increase erosion rates due to increased splash-erosion^9^. On a large scale such developments can have negative effects on sustainable water provision and cause flooding events in downstream areas^10^. Finally, the sustainability of the nomadic system of Tibetan herders and the flourishing of native flora and fauna depends on an intact vegetation cover.

Over the last decades, pronounced land-use and land-cover changes have been occurring on the QTP. A governmental reform in China in 1981 along with the First Grassland Law in 1985 facilitated the intensification of pasture management and the successive transition from the traditional nomadic pastoralism towards sedentary pastoral systems^11^. After these land-use changes, an increased number of studies reported signs of grassland degradation across the QTP^12^ with subsequent initialization of governmental restoration programs^13^.

However, due to the vast extent of the QTP, deriving a complete picture of the vegetation trends is challenging. Remote sensing datasets covering the complete QTP can be one solution. An earlier study^14^ found mostly positive trends for Normalized Difference Vegetation Index (NDVI) calculated from MODIS satellite data at a spatial grain of 1 km and for the time-period from 2000 and 2012. Another study^15^ investigated MODIS NDVI trends at 250 m spatial resolution for the time period between 2000 and 2013 and found an average increase in NDVI across the whole alpine grassland areas. They found about 75% of the study area to have rather stable NDVI trends while 25% showed significantly decreasing or increasing trends. Across the complete Plateau, climate variables could explain a notable proportion of the variation in NDVI trends. On the other hand, a recent study^16^, observed locally differing vegetation trends patterns applying MODIS data at 500 m pixel size and for the time-period between 2000 and 2013. It reported increased vegetation cover for the north-eastern Tibetan Plateau but decreasing trends for the central and western parts of the QTP. In their studies, they also found that climate variability is a more important driver for these patterns as livestock numbers.

All just cited studies used MODIS datasets with a spatial resolution of 250–1000 m for approximately the same time-period but still reported differing trends in some areas. We assume that, besides differences in the applied methodology, particularly the differing scales (pixel sizes) may explain these inconsistencies. From our field experience, we know that grassland decline often starts in comparably small patches as a result of land-use management decisions^17^. However, such declining areas may not show at larger pixel sizes due to their relatively small spatial extent, particularly if the surrounding areas show a slightly increasing trend. Furthermore, the MODIS-based products only cover a limited time-period (2000–2018) and may not be able to fully capture potential climate change influences.

This was the motivation to develop a Landsat-based vegetation trend product for the complete Tibetan Plateau at a pixel size of 30 m and for a notably increased time-span. This dataset has a high value for analyses trying to understand vegetation trends at regional and local scales. Furthermore, it can be used in various fields of research including for example wildlife ecology, livestock management, hydrology and climatology.

## Methods

### Annual Landsat-based NDVI mosaics via the Google Earth Engine

Within the Google Earth Engine environment, we used the surface reflectance collections available for the satellites Landsat 5, 7 and 8. All satellite scenes in these collections are corrected for atmospheric effects using the LEDAPS algorithms^18^ for Landsat 5 and 7 and the LaSRC algorithms for Landsat 8 (USGS Landsat Surface Reflectance Tier 1)^19^. For all images acquired between 1st of June and 30th of September of each year we masked clouds, cloud shadows and snow as identified by the CFMASK algorithm^20^ and calculated the NDVI based on the red and near infrared band. For the Landsat 8 data we applied an intercept and offset, which compensates for the different band designations between Landsat 5/7 and Landsat 8^21^. NDVI values higher and lower than 0.9 and −0.9 were masked out to remove remaining artefacts which occurred locally for example due to the Landsat 7 scan-line correction error. Subsequently, we produced annual NDVI mosaics by calculating the median NDVI value of all available scenes for the indicated time-period within a year^22^. We calculated the median instead of the mean value as it is less-affected by outlier values. Outliers can in our case occur due to pixels affected by clouds or snow which were missed during the automated masking procedures described above.

This mosaicking procedure was applied to derive three NDVI time-series, covering the time-periods between 1990–2018, 1990–2002 and 2000–2018. These time-periods were selected primarily based on data availability: the time-period between 1990–2018 is the longest time period for which a comparably dense record of Landsat observations is available for the complete QTP. The approximate time period from 2000 to 2018 has been analyzed in several earlier studies as it overlaps with the data availability of MODIS. The latter, hence, enables a comparison with earlier reported trends at lower spatial resolution. The time period is furthermore interesting as a couple of important governmental programs aiming to mitigate grassland decline (e.g., the “Grain for green” program in 1999 and the “Grazing withdrawal program” in 2003) have been launched around the year 2000^13,23^. It might hence be valuable for follow-up studies to compare the trends for the time-periods before and after the launch of these programs.

**Fig. 1:**
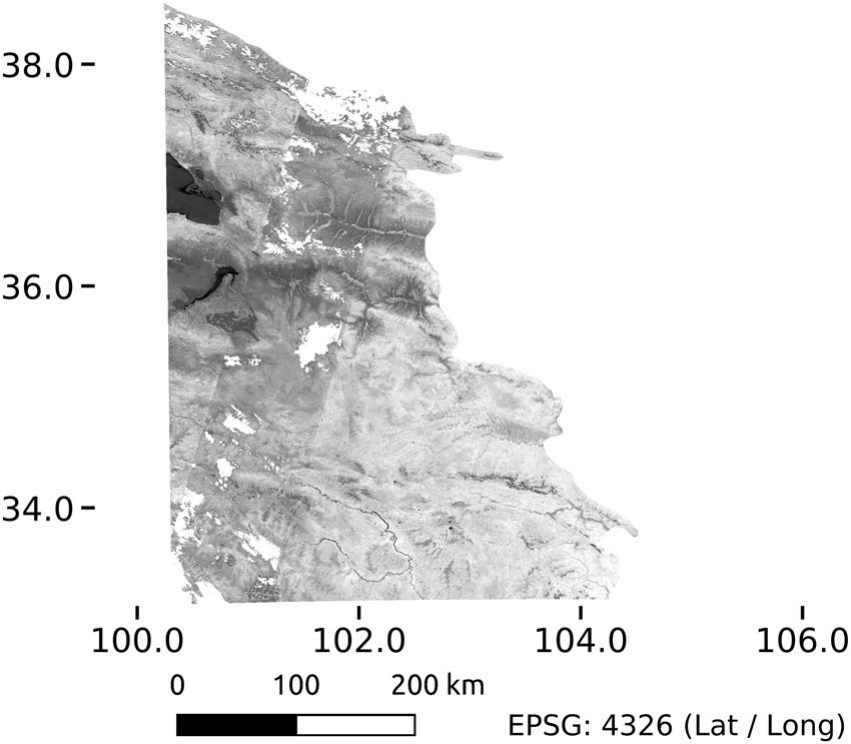
Example (Tile 2, NDVI mosaic year 1993) for an artefact caused by limited number of observation within a Landsat path which in this case lead to a notably darker (lower) NDVI value which appears as “striping” in the dataset. Mosaics affected by such artefacts were removed from the dataset.

Due to the large size of the Landsat data, the calculation of the NDVI time series was accomplished in 14 approximately equally-sized tiles. After screening preliminary results, it became clear that for some years and tiles the number of available Landsat observations was not enough to derive an artefact-free annual mosaic (an example of a mosaic with artefacts is given in Fig. 1). We hence visually checked all annual mosaics and excluded those that showed notable artefacts (mostly striping effects caused by low number of observations in one of the Landsat orbit paths). Table 1 summarizes the data availability for each tile after the screening procedure.

**Table 1:** Data availability per year and Tile.

	T1	T2	T3	T4	T5	T6	T7	T8	T9	T10	T11	T12	T13	T14
1990	√	√		√	√	√	√	√		√	√	√		√
1991	√	√	√	√	√	√	√	√	√	√	√	√	√	√
1992	√	√	√		√		√	√	√	√	√	√	√	√
1993	√			√	√	√	√	√		√	√	√	√	√
1994	√	√	√	√	√	√	√	√	√	√	√	√		√
1995			√				√			√			√	
1996	√	√	√	√	√	√	√	√	√	√	√	√	√	√
1997	√	√	√	√	√	√	√	√	√			√	√	√
1998	√	√	√	√	√	√	√	√	√	√		√		√
1999	√	√	√	√	√	√	√	√	√	√		√	√	√
2000	√	√	√	√	√	√	√	√	√	√	√	√	√	√
2001	√	√	√	√	√	√	√	√	√	√	√	√	√	√
2002	√	√	√	√	√	√	√	√	√	√	√	√	√	√
2003	√	√	√	√	√	√	√	√	√	√	√	√	√	√
2004	√	√	√	√	√	√	√	√	√	√	√	√	√	√
2005	√	√	√	√	√	√	√	√	√	√	√	√	√	√
2006	√	√	√	√	√	√	√	√	√	√	√	√	√	√
2007	√	√	√	√	√	√	√	√	√	√	√	√	√	√
2008	√	√	√	√	√	√	√	√	√	√	√	√	√	√
2009	√	√	√	√	√	√	√	√	√	√	√	√	√	√
2010		√	√	√	√	√	√		√	√	√	√	√	√
2011	√	√	√	√	√	√	√	√	√	√	√	√	√	√
2012	√		√	√	√	√		√	√	√	√	√	√	√
2013	√	√	√	√	√	√	√	√	√	√	√	√	√	√
2014	√	√	√	√		√	√	√	√	√	√	√	√	√
2015	√	√	√	√	√	√	√	√	√	√	√	√	√	√
2016	√	√	√	√	√	√	√	√	√	√	√	√	√	√
2017	√	√	√	√	√	√		√	√	√	√	√	√	√
2018	√	√	√	√	√	√	√	√	√	√	√	√	√	√

**Fig. 2:**
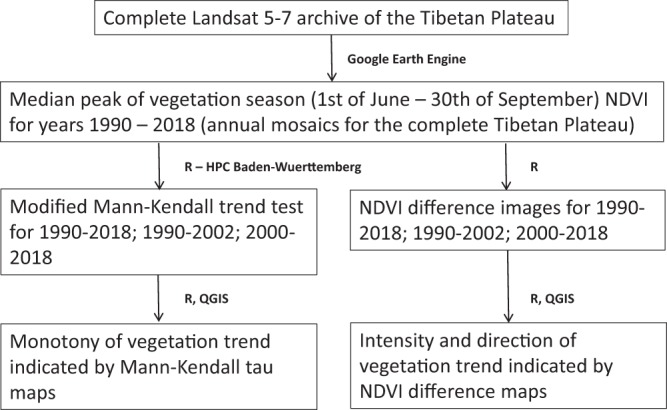
Work-flow to derive the datasets presented in this contribution.

As summarized in Fig. 2, the annual Landsat mosaics where then used to calculate Mann-Kendall trends as well as to calculate NDVI difference images as explained below.

### Modified mann-kendall trend test

The Mann-Kendall trend test tests whether a regular time series shows a monotonic (upward or downward) trend. It is a rank-based test and can hence identify both linear and non-linear trends. The null hypothesis of the Mann-Kendall trend test states that there is no trend for a time series of observations y:

1 $${H}_{0}:P({y}_{i} < {y}_{j})=\frac{1}{2}\,for\,i < j$$

While the alternative hypothesis states that there is a trend which can be positive or negative:

2 $${H}_{\alpha }:P({y}_{i} < {y}_{j})\ne \frac{1}{2}\,for\,i < j$$

The trend-statistic observed for an individual time series is calculated as Kendall’s τ which is derived with the equation:

3 $$\tau =\frac{S}{n(n-1)/2}$$

where the *S* parameter is calculated as:

4 $$S={\sum }_{k=1}^{n-1}{\sum }_{j=k+1}^{n}sgn({y}_{j}-{y}_{k})$$

and *n* denotes the number of observations in the time series.

In words, *S* is calculated by comparing the value of each time point in the time series with the value of the next step (in our case the next year) in the time series. For each comparison either a 1 (if the successive value is bigger than the tested value), a 0 (if the successive value has the same value as the tested value) or a −1 (if the successive value is smaller than the tested value) is recorded. All of these records are then summed to calculate *S*. *S* values that differ significantly from 0 indicate a monotonic trend. The term in the denominator of Eq. 3 normalizes the *S* value to form a value range between −1 and 1.

The statistical significance of the obtained *S* value is tested by calculating the test statistic *z*_*c*_.

5 $${z}_{c}=\left\{\begin{array}{ll}\frac{S-1}{\sqrt{{\sigma }^{2}}} & S > 0\\ 0 & S=0\\ \frac{S+1}{\sqrt{{\sigma }^{2}}} & S < 0\end{array}\right.$$

Where the variance σ^2^ is calculated as:

6 $${\sigma }^{2}=\frac{n(n-1)(2n+5)-{\sum }_{j=1}^{p}{t}_{j}({t}_{j}-1)(2{t}_{j}+5)}{18}$$

This function includes a correction term for tied groups (that is, groups of identical values yielding the same rank), including *t*_*j*_ denoting the number of tied values by the size of j. *z*_*c*_ follows a standard normal distribution which means that its significance can be tested. Here, we selected *α* < 0.05. A significant trend is detected, if |*z*_*c*_| > *z*_*crit*_ where *z*_*crit*_ is the value with probability *α*/2 on a standard normal distribution.

The descriptions so far refer to the standard Mann-Kendall trend test which assumes independence of the observations within the time series. First experiments applying the standard Mann-Kendal trend test revealed that the inconsistencies in the lengths of our NDVI time series (which could vary from pixel to pixel depending on how many years had a valid observation) led to visual artefacts in the trend product. These artefacts showed notably increased τ values in areas where two Landsat tiles overlapped and hence the double amount of observations was available. We hence applied a modified Mann-Kendall trend test which adapts the calculation of the variance of the *S* value to make it suitable for auto-correlated datasets and increases the comparability of the trend results between samples with varying numbers of observations. The mathematical details of these modifications can be found in the literature^23^.

As an additional step to avoid the detection of false trends, we additionally calculated the Hurst parameter to ensure that rather long-term persisting trends were detected instead of short-term fluctuation^24,25^ that could be introduced by our approach to calculate the yearly NDVI composites.

A statistically significant trend was hence assumed if firstly, the trend was significant according to the Mann-Kendall trend test; secondly, the Hurst-coefficient was significantly higher than 0.5 and thirdly, the bias corrected modified MK-trend test was significant as well.

**Fig. 3:**
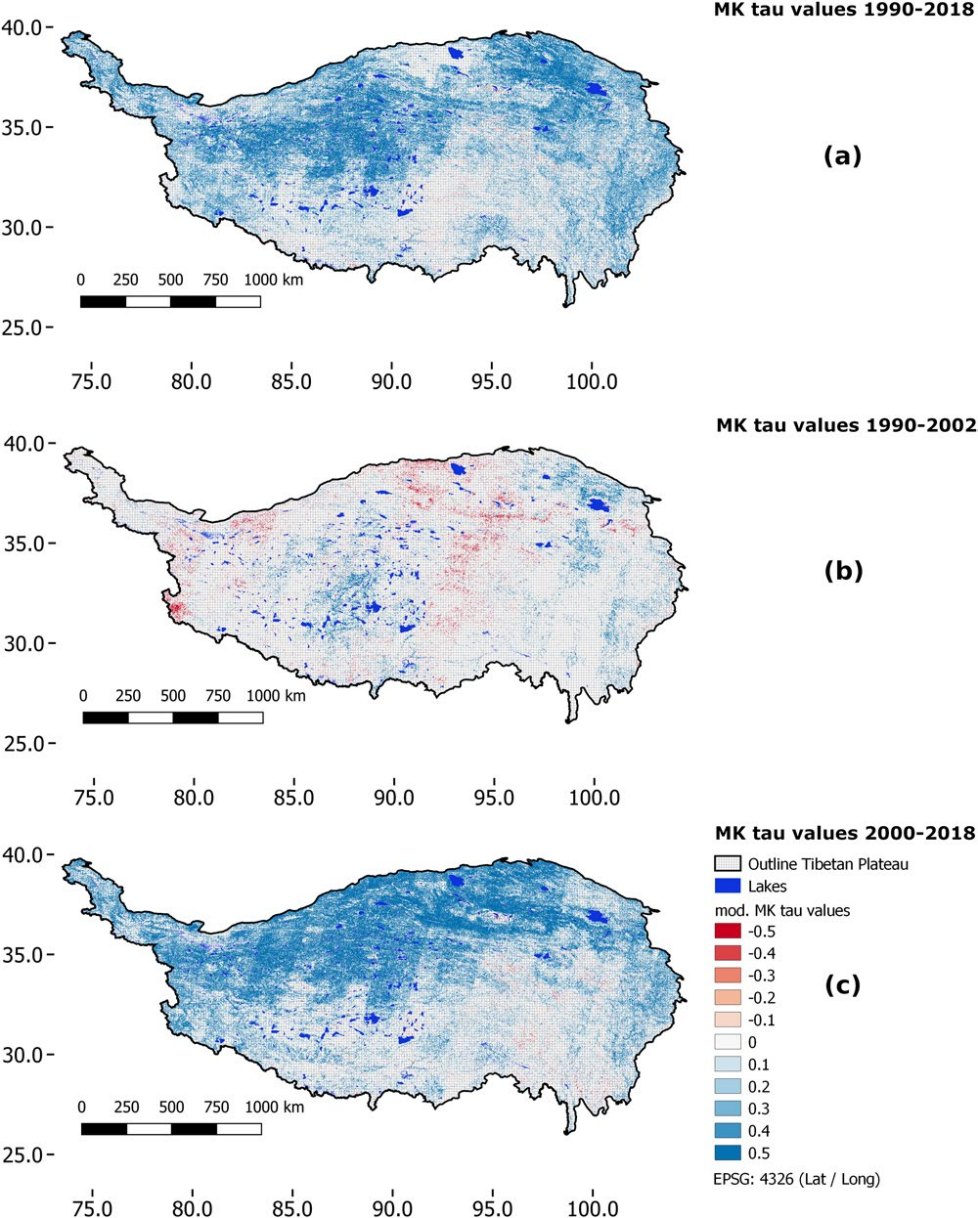
Mann-Kendall tau trend test results. Displayed are the statistically significant Mann-Kendall tau values. Panels (a–c) show the results for the time-series from 1990–2018, 1990–2002 and 2000–2018, respectively.

The final map product was then derived by first applying a significance-filter (considering the Hurst parameter and the p-value of the bias corrected MK test) on the Mann-Kendall-τ product available for the complete Tibetan Plateau and then presenting all pixels with τ-values that passed the filter in a graduated colour map^22^ (Fig. 3).

With this workflow, the visual artefacts were notably reduced but are still visible in some parts of the dataset, particularly when visualizing the results for the complete Tibetan Plateau where difference in tau-values may occur between different Landsat-orbit paths due to differing number of observations. We did not attempt to correct for this remaining patterns as these should be considered a result following data availability rather than an artefact.

### Landsat-based NDVI difference images

**Fig. 4:**
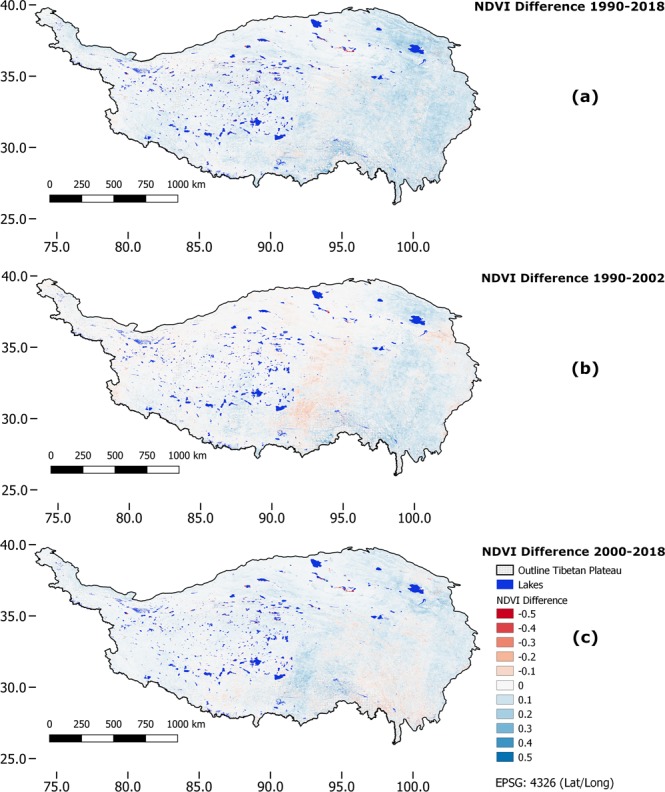
NDVI difference images. Panels (a–c) show the results for the time periods between 1990–2018, 1990–2002 and 2000–2018, respectively.

As a complement to the Mann-Kendall trend product which only describes the monotony and direction of the trend but not its intensity, we calculated NDVI difference images from the Landsat dataset. These difference images were derived by first calculating the mean NDVI of the time-periods 1990–1993, 2000–2002 and 2016–2018. The choice to average three to four scenes was a compromise between on the one hand keeping the time-period possibly short to not average out any major changes and on the other hand address the necessity to reduce the number of missing pixels in the annual mosaics and to reduce phenological effects (that might have been introduced by the Google Earth Engine procedure in years with very little cloud-free observations). Then, we derived the final NDVI difference image applying Eq. 7^22^ (Fig. 4).

7 $${NDVI}_{di\mathrm{ff}}={meanNDVI}_{{end}_{-}{of}_{-}ts}-{meanNDVI}_{{start}_{-}{of}_{-}ts}$$

The complete processing work-flow is summarized in Fig. 2. This complete work-flow was additionally used to derive the same three datasets from annual NDVI mosaics of the MODIS product MCD43A4 version 05 (BRDF adjusted 16 day reflectance product at 500 m pixel size)^22^.

### Updateability of the dataset

Based on the provided Google Earth Engine (GEE) and R codes, the suggested vegetation product can be regularly updated by simply replacing the acquisition dates in the GEE scripts and re-running the remaining code-parts. So far, this process depends on two work-steps embedded in different development environments (GEE and R). To fully automatize the data product, we will pursue the implementation of the Mann-Kendall-Trend test within the GEE or a comparable cloud environment in upcoming projects. This would allow not only accomplishing all data processing within a single environment but also to notably speed-up the calculations by making use of the computational power provided by the cloud-environment.

## Data Records

The complete dataset is available within the Figshare repository^22^. An overview over the provided datasets is given in Table 2. For an improved handling of the Landsat data, the complete Tibetan Plateau was divided into 14 tiles of approximately the same size. The first of the 10 provided sub-datasets contains the annual NDVI composites created using the procedure implemented in the Google Earth Engine for each individual tile^22^. The datasets are stored as geocoded tiff-files in geographic projection (EPSG 4326) and integer format (INT2S). The value range of each tiff-file spreads from −10000 to 10000 representing NDVI values between −1 and 1. “nan” values indicate that no valid Landsat observation was available for that pixel in the given year. Which years are available for each tile is summarized in Table 1. The individual file names follow the structure: “med_L_yyyy_poly_T.tif” where yyyy indicate the year (1990–2018) and the T the tile number (1–14).

**Table 2:** Overview of the provided datasets. All datasets are delivered in the coordinate reference system EPSG 4326.

Dataset	Format	Label	Value range
Annual Landsat NDVI mosaics	GeoTiff	med_L_yyyy_poly_T.tif	−10000 to 10000 (NDVI) NA = pixel located outside of the Tibetan Plateau
Landsat-based Mann-Kendall tau	GeoTiff	tau_sign_polyT_yyyy_xxxx	−10000 to 10000 (MK tau) −32768 = no MK test calculated because of missing data or MK test was not significant NA = pixel located outside of the Tibetan Plateau
Landsat-based NDVI Difference images	GeoTiff	NDVI_diff_yyyy_xxxx_TTT.tif	−20000 to 20000 (NDVI difference) NA = no data available at the start or the end of the time-series/pixel located outside of the Tibetan Plateau
Annual MODIS NDVI mosaics	GeoTiff	ndvi_peak_httvtt_yyyy.tif	−10000 to 10000 (NDVI) NA = pixel located outside of the Tibetan Plateau
MODIS-based Mann-Kendall tau	GeoTiff	HttVtt_tau_sign_2000_2018.tif	−10000 to 10000 (MK tau) −32768 = no MK test calculated because of missing data or MK test was not significant NA = pixel located outside of the Tibetan Plateau
MODIS-based NDVI Difference images	GeoTiff	NDVI_diff_2002_2018_httvtt.tif	−20000 to 20000 (NDVI difference) NA = no data available at the start or the end of the time-series/pixel located outside of the Tibetan Plateau

The sub-datasets 2–4 each comprise 14 geocoded tiff-files in geographic projection (EPSG 4326) that represent the results of the modified Mann-Kendall trend test for the indicated time periods and the 14 tiles^22^. Precisely, the datasets contain Kendall’s tau values (indicating the monotony of the trend) for all pixels that were found to have a statistically significant trend. All raster files contain values ranging between −10000 and 10000 indicating the corresponding Mann-Kendall tau value between −1 and 1, while a value of −32767 indicates that the time series for a given pixel was too short to calculate the trend test or that the results were non-significant and hence no result is available for that pixel. Additional ‘no data’ pixels indicate a position outside of the borders of the Tibetan Plateau, which we defined based on an official dataset of the Institute of Geographic Sciences and Natural Resources Research of the Chinese Academy of Sciences^26^. The individual file names follow the structure: “tau_sign_polyT_yyyy_xxxx” where yyyy and xxxx indicate the starting and ending years of the time series and the T the tile number (1–14).

The sub-datasets 5–7 each comprise 14 geocoded tiff-files in geographic projection (EPSG 4326) that represent the NDVI difference images for the indicated time periods^22^. All raster files contain values ranging between −20000 and 20000 indicating the change of mean NDVI from the start to the end of the corresponding time series (NDVI difference values between −2 and 2). As for the sub-datasets 2–4, additional no data pixels indicate a position outside of the borders of the Tibetan Plateau. The individual file names follow the structure: “NDVI_diff_yyyy_xxxx_T.tif” where yyyy and xxxx indicate the starting and ending years of the time series and the T the tile number (1–14).

The sub-dataset 8 comprises annual NDVI mosaics^22^ derived from MODIS product MCD43A4 version 05 (BRDF adjusted 16 day reflectance product at 500 m pixel size) following the same methodical approach described for the Landsat mosaics. These data were used for the technical validation but could also be used for independent analyses. The individual file names follow the structure: “ndvi_peak_httvtt_yyyy.tif” where yyyy indicate the year (1990–2018) and the httvtt the official code of the MODIS tile grid (e.g., h23v05)^22^.

The sub-dataset 9 comprises the same product as sub-datasets 2–4 but calculated for the MODIS dataset and only for the time period between 2000 and 2018. The individual file names follow the structure: “HttVtt_tau_sign_2000_2018.tif” where HttVtt indicate the official code of the MODIS tile grid (e.g., h23v05)^22^.

The sub-dataset 10 comprises the same product as sub-dataset 5–7 but calculated for the MODIS dataset and only for the time period between 2000 and 2018. The individual file names follow the structure: “NDVI_diff_2000_2018_httvtt.tif” where httvtt indicate the official code of the MODIS tile grid (e.g., h23v05)^22^.

The files will be accessible for download via the figshare API, which users can learn more about at https://docs.figshare.com/.”

## Technical Validation

### Comparison of the landsat NDVI mosaics with MODIS data

The quality of the vegetation trend products provided in this study directly depends on the annual Landsat mosaics derived with the Google Earth Engine procedure. One potential problem (besides pixels with missing observations) is that the average annual NDVI values could be based on a limited number of satellite observations that may have been collected rather towards the beginning or the end of the vegetation season window defined in our process. In this case, they may not fully represent the peak NDVI signal of the vegetation season of a given year. To check whether this is a notable problem in our processing chain, we compared the annual Landsat mosaics for the years 2000 to 2018 with annual NDVI mosaics of MODIS created with the identical work-flow. As MODIS has a notably higher observation frequency than Landsat (1 day repeat cycle as compared to 16 day repeat cycle of Landsat), the probability for cloud-free MODIS observations within the defined time window is 16-times higher. Correspondingly, the resulting NDVI product is expected to be very reliable and can hence serve as a reference dataset.

**Fig. 5:**
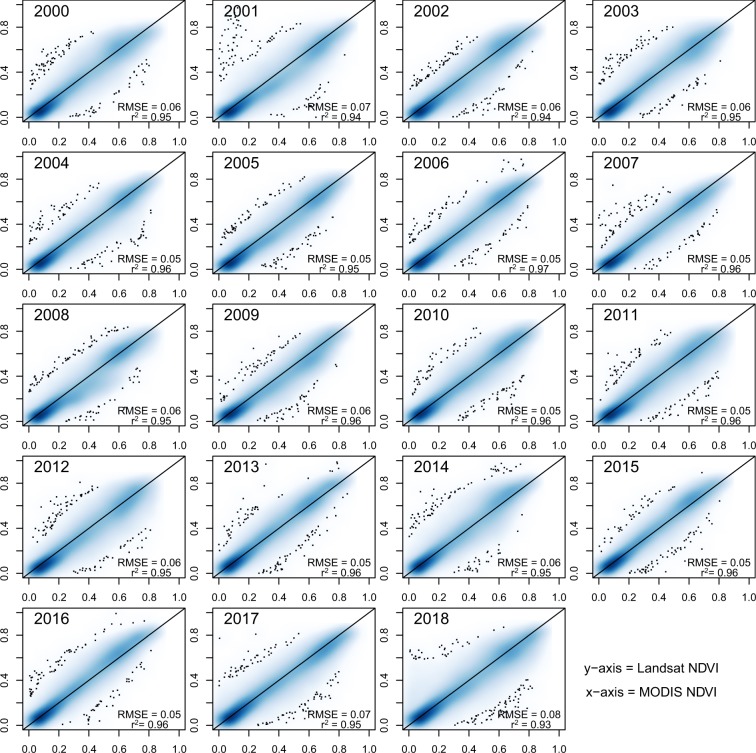
Comparison of MODIS (y-axis) and Landsat NDVI (x-axis) values for the years 2000 to 2018. The plot was created with the smoothScatter-function in R where a 2D-kernel density smoother is used to display higher number of pixels with a darker blue color. Individual black dots indicate outliers.

**Fig. 6:**
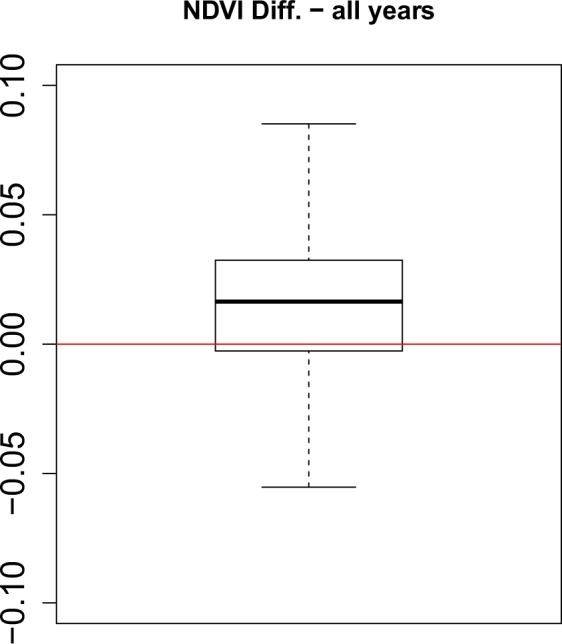
Boxplot of NDVI Differences between MODIS and Landsat for all years (245382 pixels * 19 years) (outliers are not drawn). The red line indicates the optimal zero deviance line.

To compare the Landsat-based NDVI mosaics with the MODIS-based NDVI mosaics we first created a spatial grid sized 0.25° × 0.25° covering the complete Tibetan Plateau. Then we randomly selected 100 grid cells which served as our sample for the comparison. For each of these 100 grid cells, we first cropped the corresponding MODIS and Landsat NDVI mosaics and then resampled the Landsat-subset to have the same spatial resolution (500 m pixel size) as the MODIS subset. This resulted in two raster-files with identical number of pixels. We extracted the corresponding NDVI values of each pixel and stored them into table. Such a table was created for each sampled grid cell and each year between 2000 and 2018. The tables of all 100 grid cells were used jointly to derive scatter plots between Landsat and MODIS NDVI values (Fig. 5). We additionally report the squared Pearson’s correlation coefficient and the RMSE which indicate a very high agreement between the peak of vegetation season NDVI derived by Landsat and by MODIS. Finally, a boxplot of the NDVI differences between MODIS and Landsat obtained from all 19 years shows that the vast majority of the NDVI deviances is well below 0.1 (Fig. 6). The small offset between Landsat and MODIS NDVI (median of the differences located slightly above 0) can be explained by the slightly differing band widths of the red and near-infrared channels of the two sensors.

### Further remarks

Besides this successful plausibility check, we stress that the study bases on validated Landsat reflectance products developed by NASA/USGS^17,18^. It can hence be assumed that the NDVI measurements are robust physically-based measurements that do not require further validation.

We point out that we attempted to keep our dataset as close to the original satellite data as possible. This means that we did not attempt to correct all visual irregularities that may for example exist due to differing number of observation caused by the scan-line correction error of Landsat 7. In most cases these irregularities are subtle but may still be visible in the product. We believe that this will hardly affect ecological analyses and we argue that a data-user is better-off knowing the real quality of the data as compared to work with a visually pleasing, interpolated dataset.

Finally, we stress once more that the Mann-Kendall-trend product has to be interpreted as a measure of monotony of the trend and will not give information about the intensity of the trend. A maximum Mann-Kendall value can be reached with a very small difference in absolute (NDVI) values, given that the change was very constant over time. So for many ecological applications, the Mann-Kendall information alone may not suffice.

### Visual comparison of Landsat- and MODIS-based products

**Fig. 7:**
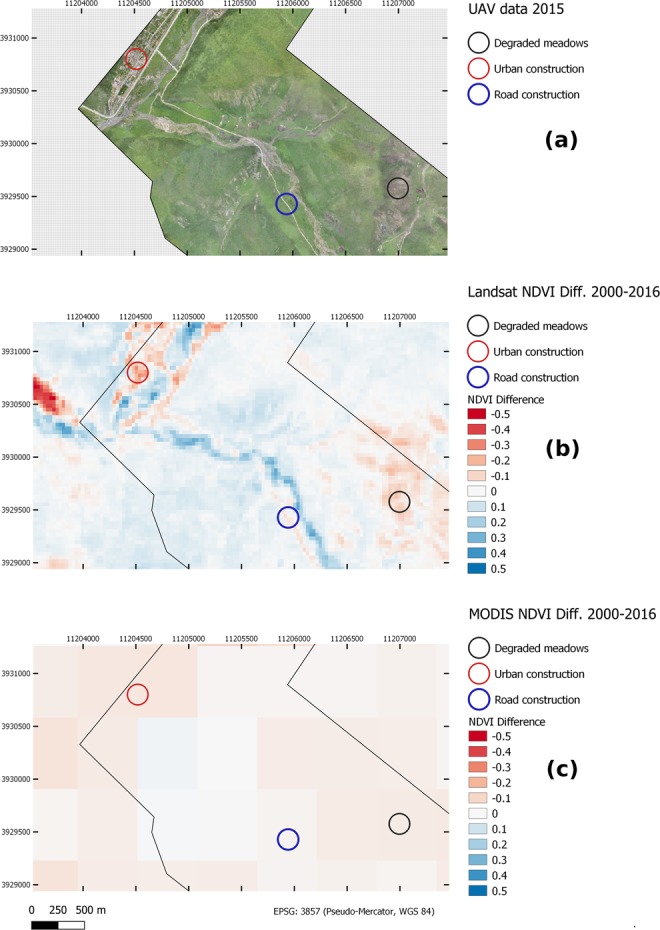
UAV data from 2015, along with NDVI difference images of MODIS and Landsat (time period between 2000 and 2016) are depicted. While in the Landsat data, detailed spatial patterns of land-cover changes can be identified, the MODIS data are too coarse to resolve the same features. In this example the MODIS data furthermore show overall notably more degrading areas than the Landsat data.

**Fig. 8:**
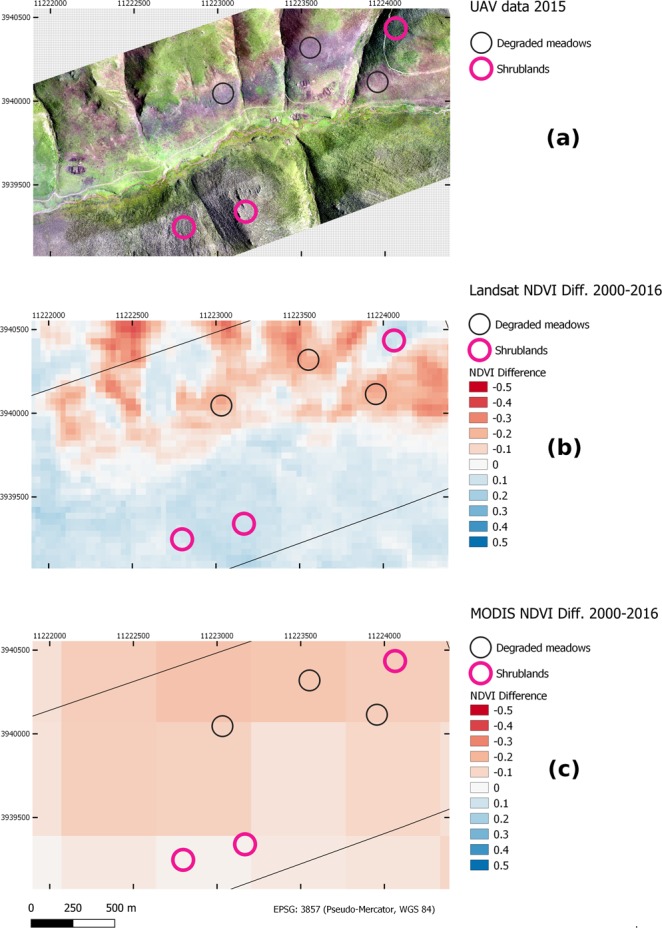
UAV data from 2015, along with the NDVI difference images of MODIS and Landsat (time period between 2000 and 2016) are depicted. The example shows degraded meadows in the Northern part of the image while shrubland prevail in the Southern part. In the Landsat difference images, the patterns of the degraded meadows are well captured by negative NDVI values while the shrublands show slightly positive values. Overall, the area covered by positive and negative values are about equal. Contrarily, the MODIS data show negative values for the complete area and cannot resolve fine spatial patterns.

The advantage of the presented dataset over datasets based on MODIS data becomes apparent in a direct comparison of the newly presented Landsat data at a pixel size 30 m and the more commonly applied MODIS data at 500 m pixel size. In Figs 7 and 8, two regional examples from the Eastern Tibetan Plateau are given for which very high resolution UAV data^27^ were available. In Fig. 7 reductions in NDVI in the time-period between 2000 and 2016 (we calculated the NDVI difference images for 2000 and 2016 for this example to better match the UAV data) can be clearly attributed to changing land-cover features such as the extension of urban areas or the construction of a road. In the MODIS dataset, these features are impossible to distinguish due to the notably coarser spatial resolution. Similarly, the second example (Fig. 8) shows spatially explicit patterns of degraded pastures (decreased NDVI) in the central to northern part of the image. In contrast the southern parts show a slight increase in NDVI, indicating an accumulation of vegetation. These spatial details cannot be detected in the MODIS product where intermediate to slight reductions in NDVI can be observed for the complete region.

## ISA-Tab metadata file

Download metadata file

## Data Availability

The source-code for Google-Earth-Engine procedure as well as R-codes for the calculation of modified Mann-Kendall-trend test and the NDVI difference images are freely available at: https://github.com/fabianfassnacht/Tibet_Landsat. The trend products were calculated using R version 3.5.1 and packages “trend (1.1.1)”, “raster (2.6–7)”, “extraDistr (1.8.10)”, “doParallel (1.0.14)”, “foreach (1.4.4)”, “matrixStats (0.53.1)”, “quantreg (5.38)”, “MCMCpack (1.4–4)”, “mcmc (0.9–5)”, “Matrix-Models 0.4–1”, “coda (0.19–2)”, “SparseM (1.77)”, “forecast (8.5)”, “stinepack (1.3)”, “rgdal (1.2–16)” and “HKProcess (0.0–2)”.
